# Supplementary material for: Risk factors for mental illness in adults with atopic eczema or psoriasis: protocol for a systematic review
Source: BMJ Open. 2020 Dec 28;10(12):e038324. doi: 10.1136/bmjopen-2020-038324 (PMC7772326; doi:10.1136/bmjopen-2020-038324)
Supplement: Supplementary data [file bmjopen-2020-038324supp001.pdf]

Supplementary Material

***Supplementary Table 1: Search strategy in MEDLINE database***

| Item number | Searches |
|---|---|
| <u>Risk factor terms</u> | |
| 1 | risk OR risk factor* OR protective factor OR predict* OR correlat* OR associate* OR aetiol* OR etiol* OR relationship OR mediat* OR mechanism* OR caus* OR path* |
| 2 | exp Risk/ |
| 3 | 1 OR 2 |
| <u>Atopic eczema terms</u> | |
| 4 | atopic dermatitis OR atopic eczema OR atopy |
| 5 | Dermatitis, Atopic/ |
| 6 | exp Eczema/ |
| 7 | 4 OR 5 OR 6 |
| <u>Psoriasis terms</u> | |
| 8 | psoriasis OR psoria* |
| 9 | pustulo* AND (palmopl* OR palmari* OR palmar) |
| 10 | exp Psoriasis/ |
| 11 | 8 OR 9 OR 10 |
| <u>Combining atopic eczema and psoriasis terms with ‘OR’</u> | |
| 12 | 7 OR 11 |
| <u>Mental illness terms</u> | |
| 13 | mental health OR mental* ill* OR mental disorder* OR affective OR anxi* OR depress* OR phobi* OR panic OR bipolar* OR schizo* OR schizophrenia OR delusion* OR psychotic* OR psychos#s |
| 14 | psychiatr* AND (ill* OR disorder OR disease*) |
| 15 | psychological* AND (ill* OR disorder OR disease* OR distress) |
| 16 | Mental Health/ |
| 17 | Exp Mental Disorders/ |
| 18 | 13 OR 14 OR 15 OR 16 OR 17 |
| <u>Combining key concepts with ‘AND’</u> | |
| 19 | 3 AND 12 AND 18 |



Adesanya EI, *et al. BMJ Open* 2020; 10:e038324. doi: 10.1136/bmjopen-2020-038324
