# Supplementary material for: Risk factors for mental illness in adults with atopic eczema or psoriasis: protocol for a systematic review
Source: BMJ Open. 2020 Dec 28;10(12):e038324. doi: 10.1136/bmjopen-2020-038324 (PMC7772326; doi:10.1136/bmjopen-2020-038324)
Supplement: Author's manuscript [file bmjopen-2020-038324.draft_revisions.pdf]

BMJ Open is committed to open peer review. As part of this commitment we make the peer review history of every article we publish publicly available.

When an article is published we post the peer reviewers' comments and the authors' responses online. We also post the versions of the paper that were used during peer review. These are the versions that the peer review comments apply to.

The versions of the paper that follow are the versions that were submitted during the peer review process. They are not the versions of record or the final published versions. They should not be cited or distributed as the published version of this manuscript.

BMJ Open is an open access journal and the full, final, typeset and author-corrected version of record of the manuscript is available on our site with no access controls, subscription charges or pay-per-view fees (http://bmjopen.bmj.com).

If you have any questions on BMJ Open's open peer review process please email info.bmjopen@bmj.com

BMJ Open

Risk factors for mental illness in adults with atopic eczema or psoriasis: protocol for a systematic review

Journal:	*BMJ Open*
Manuscript ID	bmjopen-2020-038324
Article Type:	Protocol
Date Submitted by the Author:	06-Mar-2020
Complete List of Authors:	Adesanya, Elizabeth; London School of Hygiene & Tropical Medicine, Department of Non-Communicable Disease Epidemiology<br>Schonmann, Yochai; Ben-Gurion University of the Negev Faculty of Health Sciences, Siaal Research Center for Family Medicine and Primary Care; Clalit Health Services, Department of Quality Measurements and Research<br>Hayes, Joseph; University College London, Division of Psychiatry<br>Mathur, Rohini; London School of Hygiene and Tropical Medicine, Department of Non-Communicable Disease Epidemiology<br>Mulick, Amy; London School of Hygiene and Tropical Medicine, Department of Non-Communicable Disease Epidemiology<br>Rayner, Lauren; King's College London, Department of Psychological Medicine, Institute of Psychiatry, Psychology, and Neuroscience<br>Smeeth, Liam; London School of Hygiene & Tropical Medicine, Department of Non-Communicable Disease Epidemiology<br>Smith, C; King's College London, St John's Institute of Dermatology<br>Langan, Sinead; London School of Hygiene & Tropical Medicine, Department of Non-Communicable Disease Epidemiology; Health Data Research UK<br>Mansfield, Kathryn; London School of Hygiene & Tropical Medicine, Department of Non-Communicable Disease Epidemiology
Keywords:	MENTAL HEALTH, Eczema < DERMATOLOGY, Psoriasis < DERMATOLOGY, Depression & mood disorders < PSYCHIATRY, Anxiety disorders < PSYCHIATRY, Schizophrenia & psychotic disorders < PSYCHIATRY

SCHOLARONE™
Manuscripts

I, the Submitting Author has the right to grant and does grant on behalf of all authors of the Work (as defined in the below author licence), an exclusive licence and/or a non-exclusive licence for contributions from authors who are: i) UK Crown employees; ii) where BMJ has agreed a CC-BY licence shall apply, and/or iii) in accordance with the terms applicable for US Federal Government officers or employees acting as part of their official duties; on a worldwide, perpetual, irrevocable, royalty-free basis to BMJ Publishing Group Ltd ("BMJ") its licensees and where the relevant Journal is co-owned by BMJ to the co-owners of the Journal, to publish the Work in this journal and any other BMJ products and to exploit all rights, as set out in our licence.

The Submitting Author accepts and understands that any supply made under these terms is made by BMJ to the Submitting Author unless you are acting as an employee on behalf of your employer or a postgraduate student of an affiliated institution which is paying any applicable article publishing charge ("APC") for Open Access articles. Where the Submitting Author wishes to make the Work available on an Open Access basis (and intends to pay the relevant APC), the terms of reuse of such Open Access shall be governed by a Creative Commons licence – details of these licences and which Creative Commons licence will apply to this Work are set out in our licence referred to above.

Other than as permitted in any relevant BMJ Author's Self Archiving Policies, I confirm this Work has not been accepted for publication elsewhere, is not being considered for publication elsewhere and does not duplicate material already published. I confirm all authors consent to publication of this Work and authorise the granting of this licence.

For peer review only

Risk factors for mental illness in adults with atopic eczema or psoriasis: protocol for a systematic review

Authors: Elizabeth Adesanya,[1] Yochai Schonmann,[2,3] Joseph F Hayes,[4] Rohini Mathur,[1] Amy Mulick,[1] Lauren Rayner,[5] Liam Smeeth,[1] Catherine H Smith,[6] Sinéad M Langan,[1,7] Kathryn E Mansfield[1]

Affiliations

[1] Department of Non-Communicable Disease Epidemiology, London School of Hygiene and Tropical Medicine, London, WC1E 7HT, United Kingdom

[2] Siaal Research Center for Family Medicine and Primary Care, Faculty of Health Sciences, Ben-Gurion University of the Negev, Beer-Sheva, Israel

[3] Department of Quality Measurements and Research, Clalit Health Services, Tel Aviv, Israel

[4] Division of Psychiatry, University College London, London, WC1E 6BT, United Kingdom

[5] Department of Psychological Medicine, Institute of Psychiatry, Psychology, and Neuroscience, King's College London

[6] St John's Institute of Dermatology, Guys and St Thomas' Foundation Trust and King's College London, London, SE1 9RT, United Kingdom

[7] Health Data Research UK, Gibbs Building, 215 Euston Road, London, NW1 2BE, United Kingdom

Corresponding author

Elizabeth Adesanya, elizabeth.adesanya@lshtm.ac.uk

The London School of Hygiene and Tropical Medicine, Keppel Street, London, WC1E 7HT

Key words: atopic eczema; psoriasis; risk factors; systematic review; mental illness; depression; anxiety; schizophrenia; bipolar disorder

Word count

Abstract: 294 words

Main text: 1994 (excluding title page, abstract, references, figures, tables or acknowledgements)

Tables: 3

Supplementary Tables: 1

For peer review only - http://bmjopen.bmj.com/site/about/guidelines.xhtml

ABSTRACT

Introduction:

Evidence indicates that people with the common inflammatory skin diseases atopic eczema or psoriasis are at increased risk of mental illness. However, the reasons for a relationship between skin disease and common mental disorders (i.e. depression and anxiety) or severe mental illnesses (i.e. schizophrenia, bipolar disorder and other psychoses) are unclear. Therefore, we aim to synthesise the available evidence regarding the risk factors for mental illness in adults with atopic eczema or psoriasis.

Methods and analysis:

We will conduct a systematic review of randomised controlled trials (RCTs), cohort, case-control and cross-sectional studies. We will search the following databases from inception: Medline, Embase, Global Health, Scopus, the Cochrane Library, Web of Science, Base, PsycInfo and the Global Resource of Eczema Trials (GREAT), and the grey literature databases Open Grey, PsycExtra and the New York Academy of Medicine Grey Literature Report. We will also search the bibliographies of eligible studies and relevant systematic reviews to identify additional relevant studies. Citation searching of large summary papers will be used to further identify relevant publications. Two reviewers will initially review study titles and abstracts for eligibility, followed by full text screening. We will extract data using a standardised data extraction form. We will assess the risk of bias using the Risk of bias in non-randomised studies of interventions (ROBINS-I, for observational studies) and the

For peer review only

For peer review only - http://bmjopen.bmj.com/site/about/guidelines.xhtml

revised Cochrane risk-of-bias tool (for RCTs). We will synthesise data narratively, and, if studies are sufficiently homogenous, we will consider a meta-analysis. We will assess the quality of the evidence using the Grading of Recommendations, Assessment, Development and Evaluation (GRADE) framework.

Ethics and dissemination:

Ethical approval is not required for a systematic review. Results of the review will be published in a peer-reviewed journal and disseminated through conferences.

PROSPERO registration number: CRD42020163941

For peer review only

For peer review only - http://bmjopen.bmj.com/site/about/guidelines.xhtml

ARTICLE SUMMARY

Strengths and limitations of this study

- This protocol promotes transparent review methods, enables comparison of our final review to our initial plans, minimises risk of bias, and reduces the chance of unplanned duplication.
- Our systematic review will be the first to critically evaluate studies of the risk factors for mental illness in adults with atopic eczema or psoriasis.
- We will ensure our review is comprehensive by: searching multiple scientific literature databases (including specific grey literature databases), and including a range of study types, and not limiting to English-language studies.
- However, the studies we include may use heterogenous methods and be of variable quality, which may limit our ability to calculate pooled estimates from meta-analysis and may limit our conclusions.

For peer review only

For peer review only - http://bmjopen.bmj.com/site/about/guidelines.xhtml

INTRODUCTION

Psoriasis and atopic eczema are inflammatory skin conditions associated with considerable morbidity and reduced quality of life for both sufferers and their families. Atopic eczema and psoriasis are common in the UK population – psoriasis affects between 1.3-2.6% of adults,[1] and the prevalence of atopic eczema in adults is approximately 2.5%.[2] Similarly, mental illness is common. According to the 2017 Global Burden of Disease study, mental illness is one of the leading causes of years lived with disability worldwide.[3] In England, 17% of adults have common mental disorders (CMD, including depression or anxiety).[4] Severe mental illness (SMI – including schizophrenia, bipolar affective disorder and other psychoses) affects nearly 1% of the UK population.[4] Individuals with SMI experience substantial health inequalities; they are at increased risk of serious health problems (e.g. diabetes mellitus and cardiovascular disease) and die up to 20 years earlier than the general population.[4,5]

Associations between atopic eczema or psoriasis, and mental illness are well established. Evidence suggests that people with atopic eczema or psoriasis are at increased risk of mental illness.[6-14] However, the reasons for the relationship between inflammatory skin disease and mental illness are unclear. To the best of our knowledge there are no existing systematic reviews addressing risk factors for the relationship between atopic eczema or psoriasis and mental illness in adults. Previous systematic reviews have aimed to establish summary measure of effects for the association between either atopic eczema or psoriasis, and specific mental

For peer review only - http://bmjopen.bmj.com/site/about/guidelines.xhtml

illnesses (e.g. depression), the majority have focused on the relationship between atopic eczema or psoriasis, and CMDs.[15-19] One systematic review has investigated the risk factors that mediate the association between atopic eczema and mental illness in children and adolescents only. The majority of studies in this review of children were conducted in European countries or territories. Meta-analysis of the 35 studies included in the review found that although demographic factors such as age, sex and socioeconomic status did not moderate the risk of developing mental illness in children with atopic eczema, children from predominantly minority ethnic backgrounds were more likely to be diagnosed with a mental illness in comparison to their Caucasian counterparts.[20]

The primary aim of this systematic review will be to explore, synthesise and critically evaluate the strength and quality of all available evidence on the risk factors associated with the development of mental illness (CMDs and SMIs) in adults with atopic eczema or psoriasis. If possible, we will also compare and contrast the risk factors associated with the development of mental illness in adults with atopic eczema to the risk factors in psoriasis.

For peer review only

METHODS

This study protocol adheres to the Preferred Reporting Items for Systematic review and Meta-Analysis Protocols (PRISMA-P).[21]

Patient and public involvement

Patients and/or the public were not involved in this systematic review protocol.

Eligibility criteria

We will screen studies for potential inclusion in our review according to the eligibility criteria presented in **Table 1**.

For peer review only

For peer review only - http://bmjopen.bmj.com/site/about/guidelines.xhtml

Table 1: Eligibility criteria

	Inclusion Criteria	Exclusion Criteria
Study design and characteristics	All RCTs, cohort, case-control and cross-sectional studies where an effect estimate of the risk factors for mental illness in adults with atopic eczema or psoriasis are reported or can be calculated from available data.<br><br>Studies in any language and from any geographical setting will be considered.	Ecological studies, case series studies, case reports and systematic reviews (however, relevant summary reviews will be flagged, and reference lists searched for eligible studies).<br><br>Conference proceedings, letters, editorials, opinion articles and reports (however, relevant conference proceedings/letters will be flagged to try and identify full text).
Participants	Human participants aged 18 and over with atopic eczema or psoriasis.<br><br>Studies including both adults and children where data for adults are reported separately.	Studies conducted in children or adolescents only.<br><br>Animal or cell studies.
Exposure	Risk factors for mental illness (CMD or SMI).	
Comparators	Studies must compare adults with atopic eczema or psoriasis with the risk factors of interest to adults with atopic eczema or psoriasis without the risk factors of interest.	
Outcomes	Study outcomes must be a CMD or SMI, either clinically diagnosed or self-reported with or without validated tools.	

Abbreviations: *RCT: Randomised controlled trial; CMD: Common mental disorder; SMI: Severe mental illness*

For peer review only - http://bmjopen.bmj.com/site/about/guidelines.xhtml

Information sources

We will search the following databases for relevant articles from inception: Medline, Embase, Global Health, Scopus, Cochrane Library, Web of Science, Base, PsycInfo and the Global Resource of Eczema Trials (GREAT). Both Medline and Embase capture a large amount of published literature – Medline indexes more than 5,200 journals and Embase indexes almost 8,500 journals,[22,23] – while the other databases are likely to contain appropriate papers for this review. To ensure that all relevant literature is included in the review, we will also search for grey literature in Open Grey, the New York Academy of Medicine Grey Literature Report and PsycExtra. Finally, we will search the five largest clinical trial registries – ClinicalTrials.gov, the EU Clinical Trials Register, the Japan Primary Registries Network (JPRN), ISRCTN and the Australian New Zealand Clinical Trials Registry (ANZCTR) – to identify relevant trials.[24]

Search strategy

We will search medical subject headings and free text (in titles, abstracts and keywords) for synonyms relating to three key concepts: (1) 'risk factors', (2) 'atopic eczema or psoriasis' and (3) 'mental illness' (**Table 2**). We will combine the three key concepts in the search strategy using the Boolean logic operator 'AND'. We have developed and piloted an initial search strategy in the Medline database that has been peer reviewed by a librarian (**Supplementary Table 1**), and we will adapt it appropriately for other databases. We will also manually scrutinise the reference lists and bibliographies of included studies and relevant systematic reviews to identify

For peer review only - http://bmjopen.bmj.com/site/about/guidelines.xhtml

additional papers for inclusion. Finally, we will use citation searching on large summary papers to identify any further relevant publications.

For peer review only

For peer review only - http://bmjopen.bmj.com/site/about/guidelines.xhtml

Table 2: Keywords included in the search strategy for all databases

Search term	Keywords
Risk factor terms	risk **OR** risk factor* **OR** protective factor **OR** predict* **OR** correlat* **OR** associat* **OR** aetiol* **OR** etiol* **OR** relationship **OR** mediat* **OR** mechanism* **OR** caus* **OR** path*
Atopic eczema or psoriasis terms	atopic dermatitis **OR** eczema **OR** atopy **OR** psoriasis **OR** psoria* **OR** (pustulo* AND palmopl* OR palmari* OR palmar)
Mental illness terms	mental health **OR** mental* ill* **OR** mental disorder* **OR** psychiatr* ill* **OR** psychiatr* disorder **OR** psychiatr* disease* **OR** psychological* ill* **OR** psychological* disorder* **OR** psychological* disease* **OR** affective* **OR** anxiety **OR** depression **OR** anxi* **OR** depress* **OR** phobi* **OR** panic **OR** bipolar* **OR** bipolar disorder **OR** panic disorder **OR** schizophrenia **OR** schizo* **OR** delusion* **OR** psychotic* **OR** psychos#s **OR** psychological* distress

For peer review only

Study records

Data management

A single reviewer (EA) will import all results returned from the electronic database searches into the reference management tool EndNote X9 (Clarivate Analytics, V.9.2/2019). After identifying and removing duplicate records, we will import the search results into Rayyan (a web application for systematic reviews),[25] where the integrated deduplication function will be used to identify any previously missed duplicates.

Study selection

Two reviewers (EA and YS) will independently screen the titles and abstracts of the search results for potentially relevant studies. Both reviewers will then screen the full text of all potentially relevant studies for inclusion using the eligibility criteria. Any disagreements during this process will be discussed by EA and YS, with consultation from a third reviewer (KM) if necessary. We will record and report in a flowchart the reasons for study rejection following full text screening.

Data extraction

We will develop a standardised data extraction form (to extract information described below), which will be piloted by two reviewers (EA and YS) who will extract data from the larger of either 10% or five of the eligible studies. Any disagreements between the two reviewers will be discussed, with a third reviewer (KM) available to arbitrate if required, and changes made to the data extraction form if necessary. A single

For peer review only

reviewer (EA) will complete the extraction of data for the remaining studies. We will use a modified version of the PICOS (Population, Intervention, Comparator(s), Outcome(s) and Study Design) framework to summarise data for extraction.[26] However, due to the inclusion of observational studies in our review, we will replace the term 'intervention' with 'exposure', and 'study design' will be replaced by 'study characteristics'. We will extract information for each component of the PICOS framework, in addition to study results for each study included in the review (**Table 3**).

For peer review only - http://bmjopen.bmj.com/site/about/guidelines.xhtml

Table 3: Items that will be collected using the data extraction form

Parameter	Information for extraction
Population	Participant inclusion and exclusion criteria<br>Demographic characteristics (age, sex and ethnicity distributions)<br>Sample size
Exposure	Definition and identification of individuals with the risk factor(s) of interest<br>Number of individuals with the risk factor(s) of interest
Comparator	Definition and identification of individuals without the risk factor(s) of interest<br>Number of individuals without the risk factor(s) of interest
Outcome	Definition and identification of mental illness outcome(s)<br>Number of individuals in exposed and comparison group with the outcome
Study characteristics	Bibliographic information (authors, journal, publication year, volume, page numbers, doi)<br>Study design<br>Study setting<br>Study sampling frame<br>Methods of participant recruitment<br>Aims and objectives
Study results	Unadjusted and fully adjusted effect estimates for the association between risk factors and mental illness<br>Confounders measured and adjusted for in analysis

For peer review only

For peer review only - http://bmjopen.bmj.com/site/about/guidelines.xhtml

Outcomes

Our primary outcome of interest will be mental illness in individuals with atopic eczema or psoriasis. Mental illness will be grouped into two broad categories (CMD or SMI), unless there are sufficient studies looking at specific mental illnesses (e.g. depression) when we will also explore by specific mental illness subgroup. We will include studies regardless of how they capture mental illness outcomes (i.e. we will include clinical diagnoses or self-reported mental illness established with or without validated tools).

Risk of bias assessment for individual studies

Two reviewers (EA and YS) will independently assess the risk of bias for the larger of 10%, or five, of the included studies. Any disagreements will be discussed so that a consensus can be reached. A third reviewer (KM) will be available to arbitrate if required. A single reviewer (EA) will then assess risk of bias for the remaining studies. We will use the Risk of Bias in Non-randomised Studies of Interventions (ROBINS-I) tool to assess risk of bias in observational studies.[27] ROBINS-I assesses and evaluates the risk of bias in seven different domains: (1) confounding; (2) selection of study participants; (3) classification of interventions; (4) measurement of outcomes; (5) deviations from intended interventions; (6) missing data; and (7) selective reporting of results.[27] For each observational study included, we will assess and categorise risk of bias in one of five qualitative risk of bias categories (low, moderate, serious or critical risk of bias, or no information on which to base judgement) using the signalling questions within the tool.[27] If a domain has more

For peer review only - http://bmjopen.bmj.com/site/about/guidelines.xhtml

For peer review only

than one item, we will use the highest risk of bias identified for any item within the domain to summarise the risk of bias for that domain. We will use the revised Cochrane risk-of-bias tool (RoB 2) to assess risk of bias in any RCTs included.[28] The risk-of-bias tool assesses and evaluates the risk of bias in five different domains: (1) bias from the randomisation process; (2) bias due to deviations from intended interventions; (3) bias due to missing outcome data; (4) bias in the measurement of the outcome; and (5) bias in the selection of the reported results.[28] We will classify risk of bias for each domain as: 'low risk, 'high risk', or 'some concerns. We will produce separate risk of bias tables for observational studies and RCTs, along with justifications for the decisions made.

For peer review only

For peer review only - http://bmjopen.bmj.com/site/about/guidelines.xhtml

Data synthesis and meta-bias(es)

We will synthesise our results narratively. We will describe and tabulate the results of the studies included in the review according to the study design (RCT, cohort, case-control or cross-sectional study), skin disease type (either atopic eczema or psoriasis), risk factor under investigation and outcome measure (either CMD or SMI). If possible, we will also identify risk factors that are common and distinct between atopic eczema and psoriasis. If at least two studies are sufficiently homogeneous (in terms of study design, study population, risk factor assessed and outcome), we will consider a meta-analysis to pool the effect estimates. We will use the I^2 statistic to quantify levels of statistical heterogeneity (I^2 of 0-40% may indicate negligible heterogeneity, 30-60% may indicate moderate heterogeneity, 50-90% may indicate substantial heterogeneity and 75-100% may indicate considerable heterogeneity).[24] If possible, we will also consider meta-regression to investigate whether study characteristics (e.g. study design, risk of bias, study outcome, skin disease) or the demographics of the study population (e.g. age and sex) are associated with the magnitude of effects and can explain any observed statistical heterogeneity. We will assess the risk of publication bias for the studies included in the review using funnel plots. We will use STATA V.16.0 to perform all statistical analysis.

Confidence in cumulative evidence

We will use the Grading of Recommendations, Assessment, Development and Evaluation (GRADE) framework to evaluate and summarise the quality of cumulative evidence for each broad outcome (CMD or SMI) and risk factor pair.[29] If more than

For peer review only - http://bmjopen.bmj.com/site/about/guidelines.xhtml

one study is identified for a specific subtype of a CMD or SMI (such as depression or schizophrenia) and a specific risk factor, we will use GRADE to summarise the quality of evidence for that subtype. We will categorise the strength of evidence into four qualitative categories: ‘high’, ‘moderate’, ‘low’ or ‘very low’. The quality of evidence for included studies will be upgraded if there is a large magnitude of effect or a dose-response gradient.[29] The quality of evidence will be rated down if there is a high risk of bias, imprecision in the study estimate, a high probability of publication bias or inconsistent results.[29] We will present the judgments made during this process in a ‘Summary of Findings’ table.

For peer review only

ETHICS AND DISSEMINATION

As this study is a systematic review that does not involve human participation, we do not require ethical approval. We will disseminate the results of this review by publishing in an open access, peer-reviewed journal and presenting at conferences. We will document any important amendments and protocol deviations, along with justifications, and publish them as an appendix in the final review.

For peer review only

For peer review only - http://bmjopen.bmj.com/site/about/guidelines.xhtml

REFERENCES

1. Parisi R, Symmons DP, Griffiths CE et al. Global Epidemiology of Psoriasis: A Systematic Review of Incidence and Prevalence. *J Invest Dermatol*. 2013; 133(2):377-85. doi: 10.1038/jid.2012.339
2. Barbarot S, Auziere S, Gadkari A et al. Epidemiology of atopic dermatitis in adults: Results from an international survey. *Allergy*. 2018;73(6):1284-1293. doi: 10.1111/all.13401
3. James S, Abate D, Abate K et al. Global, regional, and national incidence, prevalence, and years lived with disability for 354 diseases and injuries for 195 countries and territories, 1990–2017: a systematic analysis for the Global Burden of Disease Study 2017. *Lancet*. 2018;392(10159):1789-1858. doi: 10.1016/S0140-6736(18)32279-7
4. NHS Digital. *Adult Psychiatric Morbidity Survey: Survey of Mental Health and Wellbeing, England, 2014*. Available from: https://digital.nhs.uk/data-and-information/publications/statistical/adult-psychiatric-morbidity-survey/adult-psychiatric-morbidity-survey-survey-of-mental-health-and-wellbeing-england-2014#key-facts [Accessed 21st January 2020]
5. Chesney E, Goodwin G, Fazel S. Risks of all-cause and suicide mortality in mental disorders: a meta-review. *World Psychiatry*. 2014;13(2):153-160. doi: 10.1002/wps.20128.
6. Eckert L, Gupta S, Amand C et al. Impact of atopic dermatitis on health-related quality of life and productivity in adults in the United States: An analysis using the National Health and Wellness Survey. *J Am Acad Dermatol*. 2017;77:274–279.e3. doi: 10.1016/j.jaad.2017.04.019
7. Klokk M, Gotestam KG, Mykletun A. Factors accounting for the association between anxiety and depression, and eczema: The Hordaland health study (HUSK). *BMC Dermatol*. 2010;10:3. doi: 10.1186/1471-5945-10-3
8. Schmitt J, Romanos M, Pfennig A et al. Psychiatric comorbidity in adult eczema. *Br J Dermatol*. 2009;161(4):878–83. doi: 10.1111/j.1365-2133.2009.09309

For peer review only - http://bmjopen.bmj.com/site/about/guidelines.xhtml

9. Ferreira BR, Pio-Abreu JL, Reis JP et al. Analysis of the prevalence of mental disorders in psoriasis: The relevance of psychiatric assessment in dermatology. *Psychiatr Danub*. 2017;29(4):401–6. doi: 10.24869/psyd.2017.401.

10. Kurd SK, Troxel AB, Crits-Cristoph P et al. The Risk of Depression, Anxiety, and Suicidality in Patients With Psoriasis. *Arch Dermatol*. 2010;146(8):891–5. doi: 10.1001/archdermatol.2010.186

11. Tu HP, Yu CL, Lan CCE et al. Prevalence of schizophrenia in patients with psoriasis: a nationwide study. *Dermatologica Sin*. 2017;35(1):1–6. doi: 10.1016/j.dsi.2016.07.005

12. Schonmann Y, Mansfield K, Hayes J et al. Atopic Eczema in Adulthood and Risk of Depression and Anxiety: A Population-Based Cohort Study. *J Allergy Clin Immunol Pract*. 2020;8(1):248-257. doi: 10.1016/j.jaip.2019.08.030

13. Drucker AM, Thiruchelvam D, Redelmeier DA. Eczema and subsequent suicide: a matched case–control study. *BMJ Open*. 2018;8:e023776. doi: 10.1136/bmjopen-2018-023776

14. Sandhu JK, Wu KK, Bui T et al. Association Between Atopic Dermatitis and Suicidality: A Systematic Review and Meta-analysis. *JAMA Dermatol.* 2019;155(2):178–187. doi:10.1001/jamadermatol.2018.4566

15. Bao Q, Chen L, Lu Z et al. Association between eczema and risk of depression: A systematic review and meta-analysis of 188,495 participants*. J Affect Disord*. 2018; 238:458–64.doi: 10.1016/j.jad.2018.05.007

16. Rønnstad ATM, Halling-Overgaard A-S, Hamann CR et al. Association of atopic dermatitis with depression, anxiety, and suicidal ideation in children and adults: A systematic review and meta-analysis. *J Am Acad Dermatol*. 2018;79(3):448-456.e30. doi: 10.1016/j.jaad.2018.03.017

17. Dowlatshahi EA, Wakkee M, Arends LR et al. The prevalence and odds of depressive symptoms and clinical depression in psoriasis patients: A systematic review and meta-analysis. *J Invest Dermatol*. 2014;134(6):1542–1551. doi: 10.1038/jid.2013.508

For peer review only - http://bmjopen.bmj.com/site/about/guidelines.xhtml

18. Ferreira B.I.R.C, Abreu J.L.P., Dos Reis J.P.G. et al. Psoriasis and associated psychiatric disorders: A systematic review on etiopathogenesis and clinical correlation. *J Clin Aesthet Dermatol*. 2016;9(6):36–43. Available from: https://www.ncbi.nlm.nih.gov/pmc/articles/PMC4928455/ [Accessed 21st January 2020]

19. Patel K, Immaneni S, Singam V et al. Association between atopic dermatitis, depression, and suicidal ideation: A systematic review and meta-analysis. *J Am Acad Dermatol*. 2019; 80(2):402-410. doi: 10.1016/j.jaad.2018.08.063

20. Xie Q, Dai X, Tang X et al. Risk of Mental Disorders in Children and Adolescents with Atopic Dermatitis: A Systematic Review and Meta-Analysis. *Front Psychol*. 2019; 10:1773. doi: 10.3389/fpsyg.2019.01773

21. Moher D, Shamseer L, Clarke M et al. Preferred reporting items for systematic review and meta-analysis protocols (PRISMA-P) 2015 statement. *Syst Rev*. 2015;4(1):1. doi: https://doi.org/10.1186/2046-4053-4-1

22. U.S National Library of Medicine. *Fact sheet: MEDLINE*. Available from: https://www.nlm.nih.gov/lstrc/jsel.html. [Accessed 27th January 2020]

23. Elsevier. *EMBASE: Coverage and Content*. Available from: https://www.elsevier.com/solutions/embase-biomedical-research/embase-coverage-and-content. [Accessed 27th January 2020]

24. Huser V, Cimino JJ. Evaluating adherence to the International Committee of Medical Journal Editors' policy of mandatory, timely clinical trial registration. J Am Med Inform Assoc. 2013; 20(e1): e169-e174. doi: 10.1136/amiajnl-2012-001501

25. Ouzzani M, Hammady H, Fedorowicz Z et al. Rayyan – a web and mobile app for systematic reviews. Syst Rev. 2016;5(1):210. doi: 10.1186/s13643-016-0384-4

26. Higgins J, Green S. *Cochrane Handbook for Systematic Reviews of Interventions: Version 5.1.0 (updated March 2011)*. 2011. Available from: www.handbook-5-1.cochrane.org [Accessed 21st January 2020]

For peer review only - http://bmjopen.bmj.com/site/about/guidelines.xhtml

27. Sterne JAC, Hernán M, Reeves B et al. ROBINS-I: a tool for assessing risk of bias in non-randomised studies of interventions. *BMJ*. 2016;355:i4919. doi: 10.1136/bmj.i4919

28. Sterne JAC, Savovic J, Page MJ et al. RoB 2: a revised tool for assessing risk of bias in randomised trials. *BMJ*. 2019;366:l4898. doi: 10.1136/bmj.l4898.

29. Guyatt G, Oxman A, Vist G et al. GRADE: an emerging consensus on rating quality of evidence and strength of recommendations. BMJ. 2008; 336(7650):924-6. doi: 10.1136/bmj.39489.470347.AD

For peer review only

ACKNOWLEDGEMENTS AND DECLARATIONS

Contributions

EA, SL and KM had the original idea for the review. All authors were involved in the design of the study. EA wrote the first draft of the protocol. All authors contributed to further drafts and approved the final manuscript. Kate Perris peer reviewed the search strategy.

Funding

EA was funded by a British Skin Foundation (BSF) PhD studentship (Reference: 024/S/18). SML was funded by a Wellcome Trust Senior Clinical Fellowship in Science (Reference: 205039/Z/16/Z).

Competing interests

None declared.

For peer review only

Supplementary Material

Supplementary Table 1: Search strategy in MEDLINE database

Item number	Searches
Risk factor terms	
1	risk OR risk factor* OR protective factor OR predict* OR correlat* OR associat* OR aetiol* OR etiol* OR relationship OR mediat* OR mechanism* OR cause* OR causal* OR causation* OR causative* OR pathway*
2	exp Risk/
3	1 OR 2
Atopic eczema terms	
4	atopic dermatitis OR atopic eczema OR eczema OR atopy
5	Dermatitis, Atopic/
6	exp Eczema/
7	4 OR 5 OR 6
Psoriasis terms	
8	psoriasis OR psoria*
9	pustulo* AND (palmopl* OR palmari* OR palmar)
10	exp Psoriasis/
11	8 OR 9 OR 10
Combining atopic eczema and psoriasis terms with 'OR'	
12	7 OR 11
Mental illness terms	
13	mental health OR mental* ill* OR mental disorder* OR affective OR anxiety OR anxi* OR depression OR depress* OR phobi* OR panic OR bipolar* OR schizo* OR schizophrenia OR delusion* OR psychotic* OR psychos#s
14	psychiatr* ill* OR psychiatr* disorder OR psychiatr* disease
15	psychological* ill* OR psychological* disorder OR psychological* disease* OR psychological* distress
16	Mental Health/
17	Exp Mental Disorders/
18	13 OR 14 OR 15 OR 16 OR 17
Combining key concepts with 'AND'	
19	3 AND 12 AND 18

For peer review only

Reporting checklist for protocol of a systematic review.

Based on the PRISMA-P guidelines.

Instructions to authors

Complete this checklist by entering the page numbers from your manuscript where readers will find each of the items listed below.

Your article may not currently address all the items on the checklist. Please modify your text to include the missing information. If you are certain that an item does not apply, please write "n/a" and provide a short explanation.

Upload your completed checklist as an extra file when you submit to a journal.

In your methods section, say that you used the PRISMA-Preporting guidelines, and cite them as:

Moher D, Shamseer L, Clarke M, Ghersi D, Liberati A, Petticrew M, Shekelle P, Stewart LA. Preferred Reporting Items for Systematic Review and Meta-Analysis Protocols (PRISMA-P) 2015 statement. Syst Rev. 2015;4(1):1.

For peer review only

		Reporting Item	Page Number
**Title**			
Identification	#1a	Identify the report as a protocol of a systematic review	1
Update	#1b	If the protocol is for an update of a previous systematic review, identify as such	n/a

**Registration**			
	#2	If registered, provide the name of the registry (such as PROSPERO) and registration number	2
**Authors**			
Contact	#3a	Provide name, institutional affiliation, e-mail address of all protocol authors; provide physical mailing address of corresponding author	1
Contribution	#3b	Describe contributions of protocol authors and identify the guarantor of the review	18
**Amendments**			
	#4	If the protocol represents an amendment of a previously completed or published protocol, identify as such and list changes; otherwise, state plan for documenting important protocol amendments	14
**Support**			
Sources	#5a	Indicate sources of financial or other support for the review	18
Sponsor	#5b	Provide name for the review funder and / or sponsor	18
Role of sponsor or funder	#5c	Describe roles of funder(s), sponsor(s), and / or institution(s), if any, in developing the protocol	18
**Introduction**			

For peer review only

Rationale	#6	Describe the rationale for the review in the context of what is already known	4-5
Objectives	#7	Provide an explicit statement of the question(s) the review will address with reference to participants, interventions, comparators, and outcomes (PICO)	4-5
**Methods**			
Eligibility criteria	#8	Specify the study characteristics (such as PICO, study design, setting, time frame) and report characteristics (such as years considered, language, publication status) to be used as criteria for eligibility for the review	7 (Table 1)
Information sources	#9	Describe all intended information sources (such as electronic databases, contact with study authors, trial registers or other grey literature sources) with planned dates of coverage	8
Search strategy	#10	Present draft of search strategy to be used for at least one electronic database, including planned limits, such that it could be repeated	8-9, Table 2 and Supplementary table 1
Study records - data management	#11a	Describe the mechanism(s) that will be used to manage records and data throughout the review	10
Study records - selection process	#11b	State the process that will be used for selecting studies (such as two independent reviewers)	10

For peer review only

		through each phase of the review (that is, screening, eligibility and inclusion in meta-analysis)	
Study records - data collection process	#11c	Describe planned method of extracting data from reports (such as piloting forms, done independently, in duplicate), any processes for obtaining and confirming data from investigators	10
Data items	#12	List and define all variables for which data will be sought (such as PICO items, funding sources), any pre-planned data assumptions and simplifications	11 (Table 3)
Outcomes and prioritization	#13	List and define all outcomes for which data will be sought, including prioritization of main and additional outcomes, with rationale	12
Risk of bias in individual studies	#14	Describe anticipated methods for assessing risk of bias of individual studies, including whether this will be done at the outcome or study level, or both; state how this information will be used in data synthesis	12
Data synthesis	#15a	Describe criteria under which study data will be quantitatively synthesised	13
Data synthesis	#15b	If data are appropriate for quantitative synthesis, describe planned summary measures, methods of handling data and methods of combining data from studies, including any planned exploration of consistency (such as I2, Kendall's τ)	13

For peer review only

Data synthesis	#15c	Describe any proposed additional analyses (such as sensitivity or subgroup analyses, meta-regression)	13
Data synthesis	#15d	If quantitative synthesis is not appropriate, describe the type of summary planned	13
Meta-bias(es)	#16	Specify any planned assessment of meta-bias(es) (such as publication bias across studies, selective reporting within studies)	13
Confidence in cumulative evidence	#17	Describe how the strength of the body of evidence will be assessed (such as GRADE)	13

None The PRISMA-P checklist is distributed under the terms of the Creative Commons Attribution License CC-BY 4.0. This checklist can be completed online using https://www.goodreports.org/, a tool made by the EQUATOR Network in collaboration with Penelope.ai

For peer review only

Risk factors for mental illness in adults with atopic eczema or psoriasis: protocol for a systematic review

Journal:	*BMJ Open*
Manuscript ID	bmjopen-2020-038324.R1
Article Type:	Protocol
Date Submitted by the Author:	28-Sep-2020
Complete List of Authors:	Adesanya, Elizabeth; London School of Hygiene & Tropical Medicine, Department of Non-Communicable Disease Epidemiology<br>Schonmann, Yochai; Ben-Gurion University of the Negev Faculty of Health Sciences, Siaal Research Center for Family Medicine and Primary Care; Clalit Health Services, Department of Quality Measurements and Research<br>Hayes, Joseph; University College London, Division of Psychiatry<br>Mathur, Rohini; London School of Hygiene and Tropical Medicine, Department of Non-Communicable Disease Epidemiology<br>Mulick, Amy; London School of Hygiene and Tropical Medicine, Department of Non-Communicable Disease Epidemiology<br>Rayner, Lauren; King's College London, Department of Psychological Medicine, Institute of Psychiatry, Psychology, and Neuroscience<br>Smeeth, Liam; London School of Hygiene & Tropical Medicine, Department of Non-Communicable Disease Epidemiology<br>Smith, C; King's College London, St John's Institute of Dermatology<br>Langan, Sinead; London School of Hygiene & Tropical Medicine, Department of Non-Communicable Disease Epidemiology; Health Data Research UK<br>Mansfield, Kathryn; London School of Hygiene & Tropical Medicine, Department of Non-Communicable Disease Epidemiology
**Primary Subject Heading**:	Epidemiology
Secondary Subject Heading:	Dermatology, Epidemiology, Mental health
Keywords:	MENTAL HEALTH, Eczema < DERMATOLOGY, Psoriasis < DERMATOLOGY, Depression & mood disorders < PSYCHIATRY, Anxiety disorders < PSYCHIATRY, Schizophrenia & psychotic disorders < PSYCHIATRY

I, the Submitting Author has the right to grant and does grant on behalf of all authors of the Work (as defined in the below author licence), an exclusive licence and/or a non-exclusive licence for contributions from authors who are: i) UK Crown employees; ii) where BMJ has agreed a CC-BY licence shall apply, and/or iii) in accordance with the terms applicable for US Federal Government officers or employees acting as part of their official duties; on a worldwide, perpetual, irrevocable, royalty-free basis to BMJ Publishing Group Ltd ("BMJ") its licensees and where the relevant Journal is co-owned by BMJ to the co-owners of the Journal, to publish the Work in this journal and any other BMJ products and to exploit all rights, as set out in our licence.

The Submitting Author accepts and understands that any supply made under these terms is made by BMJ to the Submitting Author unless you are acting as an employee on behalf of your employer or a postgraduate student of an affiliated institution which is paying any applicable article publishing charge ("APC") for Open Access articles. Where the Submitting Author wishes to make the Work available on an Open Access basis (and intends to pay the relevant APC), the terms of reuse of such Open Access shall be governed by a Creative Commons licence – details of these licences and which Creative Commons licence will apply to this Work are set out in our licence referred to above.

Other than as permitted in any relevant BMJ Author's Self Archiving Policies, I confirm this Work has not been accepted for publication elsewhere, is not being considered for publication elsewhere and does not duplicate material already published. I confirm all authors consent to publication of this Work and authorise the granting of this licence.

For peer review only

Risk factors for mental illness in adults with atopic eczema or psoriasis: protocol for a systematic review

Authors: Elizabeth Adesanya,[1] Yochai Schonmann,[2,3] Joseph F Hayes,[4] Rohini Mathur,[1] Amy Mulick,[1] Lauren Rayner,[5] Liam Smeeth,[1] Catherine H Smith,[6] Sinéad M Langan,[1,7] Kathryn E Mansfield[1]

Affiliations

[1] Department of Non-Communicable Disease Epidemiology, London School of Hygiene and Tropical Medicine, London, WC1E 7HT, United Kingdom

[2] Siaal Research Center for Family Medicine and Primary Care, Faculty of Health Sciences, Ben-Gurion University of the Negev, Beer-Sheva, Israel

[3] Department of Quality Measurements and Research, Clalit Health Services, Tel Aviv, Israel

[4] Division of Psychiatry, University College London, London, WC1E 6BT, United Kingdom

[5] Department of Psychological Medicine, Institute of Psychiatry, Psychology, and Neuroscience, King's College London

[6] St John's Institute of Dermatology, Guys and St Thomas' Foundation Trust and King's College London, London, SE1 9RT, United Kingdom

[7] Health Data Research UK, Gibbs Building, 215 Euston Road, London, NW1 2BE, United Kingdom

Corresponding author

Elizabeth Adesanya, elizabeth.adesanya@lshtm.ac.uk

The London School of Hygiene and Tropical Medicine, Keppel Street, London, WC1E 7HT

Key words: atopic eczema; psoriasis; risk factors; systematic review; mental illness; depression; anxiety; schizophrenia; bipolar disorder

Word count

Abstract: 285 words

Main text: 2072 (excluding title page, abstract, references, figures, tables or acknowledgements)

Tables: 3

Supplementary Tables: 1

For peer review only - http://bmjopen.bmj.com/site/about/guidelines.xhtml

ABSTRACT

Introduction:

Evidence indicates that people with the common inflammatory skin diseases atopic eczema or psoriasis are at increased risk of mental illness. However, the reasons for a relationship between skin disease and common mental disorders (i.e. depression and anxiety) or severe mental illnesses (i.e. schizophrenia, bipolar disorder and other psychoses) are unclear. Therefore, we aim to synthesise the available evidence regarding the risk factors for mental illness in adults with atopic eczema or psoriasis.

Methods and analysis:

We will conduct a systematic review of randomised controlled trials (RCTs), cohort, case-control and cross-sectional studies. We will search the following databases from inception to March 2020: Medline, Embase, Global Health, Scopus, the Cochrane Library, Web of Science, Base, PsycInfo and the Global Resource of Eczema Trials (GREAT), and the grey literature databases Open Grey, PsycExtra and the New York Academy of Medicine Grey Literature Report. We will also search the bibliographies of eligible studies and relevant systematic reviews to identify additional relevant studies. Citation searching of large summary papers will be used to further identify relevant publications. Two reviewers will initially review study titles and abstracts for eligibility, followed by full text screening. We will extract data using a standardised data extraction form. We will assess the risk of bias of included studies using the Quality in Prognosis Studies (QUIPS) tool. We will synthesise data

For peer review only - http://bmjopen.bmj.com/site/about/guidelines.xhtml

narratively, and, if studies are sufficiently homogenous, we will consider a meta-analysis. We will assess the quality of the evidence using the Grading of Recommendations, Assessment, Development and Evaluation (GRADE) framework.

Ethics and dissemination:

Ethical approval is not required for a systematic review. Results of the review will be published in a peer-reviewed journal and disseminated through conferences.

PROSPERO registration number: CRD42020163941

For peer review only

For peer review only - http://bmjopen.bmj.com/site/about/guidelines.xhtml

ARTICLE SUMMARY

Strengths and limitations of this study

- This protocol promotes transparent review methods, enables comparison of our final review to our initial plans, minimises risk of bias, and reduces the chance of unplanned duplication.
- Our systematic review will be the first to critically evaluate studies of the risk factors for mental illness in adults with atopic eczema or psoriasis.
- We will ensure our review is comprehensive by: searching multiple scientific literature databases (including specific grey literature databases), and including a range of study types, and not limiting to English-language studies.
- However, the studies we include may use heterogenous methods and be of variable quality, which may limit our ability to calculate pooled estimates from meta-analysis and may limit our conclusions.

For peer review only

For peer review only - http://bmjopen.bmj.com/site/about/guidelines.xhtml

INTRODUCTION

Psoriasis and atopic eczema are inflammatory skin conditions associated with considerable morbidity and reduced quality of life for both sufferers and their families. Atopic eczema and psoriasis are common in the UK population – psoriasis affects between 1.3-2.6% of adults,[1] and the prevalence of atopic eczema in adults is approximately 2.5%.[2] Similarly, mental illness is common. According to the 2017 Global Burden of Disease study, mental illness is one of the leading causes of years lived with disability worldwide.[3] In England, 17% of adults have common mental disorders (CMD, including depression or anxiety).[4] Severe mental illness (SMI – including schizophrenia, bipolar affective disorder and other psychoses) affects nearly 1% of the UK population.[4] Individuals with SMI experience substantial health inequalities; they are at increased risk of serious health problems (e.g. diabetes mellitus and cardiovascular disease) and die up to 20 years earlier than the general population.[4,5]

Associations between atopic eczema or psoriasis, and mental illness are well established. Evidence suggests that people with atopic eczema or psoriasis are at increased risk of mental illness.[6-14] The temporal sequence of the associations between skin disease and mental illness are also well recognised, with evidence suggesting that atopic eczema or psoriasis precede mental illness diagnosis.[10,12] However, the reasons for the relationship between inflammatory skin disease and mental illness are unclear. To the best of our knowledge there are no existing systematic reviews addressing risk factors for the relationship between atopic

For peer review only - http://bmjopen.bmj.com/site/about/guidelines.xhtml

eczema or psoriasis and mental illness in adults. Previous systematic reviews have aimed to establish summary measure of effects for the association between either atopic eczema or psoriasis, and specific mental illnesses (e.g. depression), the majority have focused on the relationship between atopic eczema or psoriasis, and CMDs.[15-19] One systematic review has investigated the risk factors that mediate the association between atopic eczema and mental illness in children and adolescents only. The majority of studies in this review of children were conducted in European countries or territories. Meta-analysis of the 35 studies included in the review found that although demographic factors such as age, sex and socioeconomic status did not moderate the risk of developing mental illness in children with atopic eczema, children from predominantly minority ethnic backgrounds were more likely to be diagnosed with a mental illness in comparison to their Caucasian counterparts.[20]

The primary aim of this systematic review will be to explore, synthesise and critically evaluate the strength and quality of all available evidence on the risk factors associated with the development of mental illness (CMDs and SMIs) in adults with atopic eczema or psoriasis. If possible, we will also compare and contrast the risk factors associated with the development of mental illness in adults with atopic eczema to the risk factors in psoriasis. In the context of this systematic review, we will use the term ‘risk factor’ to refer to variables associated with an increased risk of mental illness in individuals with atopic eczema or psoriasis.

For peer review only - http://bmjopen.bmj.com/site/about/guidelines.xhtml

METHODS

This study protocol adheres to the Preferred Reporting Items for Systematic review and Meta-Analysis Protocols (PRISMA-P).[21]

Patient and public involvement

Patients and/or the public were not involved in this systematic review protocol.

Eligibility criteria

We will screen studies for potential inclusion in our review according to the eligibility criteria presented in **Table 1**.

For peer review only

For peer review only - http://bmjopen.bmj.com/site/about/guidelines.xhtml

Table 1: Eligibility criteria

	Inclusion Criteria	Exclusion Criteria
Study design and characteristics	All RCTs, cohort, case-control and cross-sectional studies where an effect estimate (i.e. ratio or difference measures) of the risk factors for mental illness in adults with atopic eczema or psoriasis are reported.<br><br>Studies in any language and from any geographical setting will be considered.	Ecological studies, case series studies, case reports and systematic reviews (however, relevant summary reviews will be flagged, and reference lists searched for eligible studies).<br><br>Studies where correlates (without a measure of effect) have been calculated to estimate the association between a risk factor and mental illness in adults with atopic eczema or psoriasis.<br><br>Conference proceedings, letters, editorials, opinion articles and reports (however, relevant conference proceedings/letters will be flagged to try and identify full text).
Participants	Human participants aged 18 and over with atopic eczema or psoriasis.<br><br>Studies including both adults and children where data for adults are reported separately.	Studies conducted in children or adolescents only.<br><br>Animal or cell studies.
Exposure	Risk factors for mental illness (CMD or SMI).	
Comparators	Studies must compare adults with atopic eczema or psoriasis with the risk factors of interest to adults with atopic eczema or psoriasis without the risk factors of interest.	
Outcomes	Study outcomes must be a CMD or SMI, either clinically diagnosed or self-reported with or without validated tools.	

For peer review only

Abbreviations: *RCT: Randomised controlled trial; CMD: Common mental disorder; SMI: Severe mental illness*

For peer review only

For peer review only - http://bmjopen.bmj.com/site/about/guidelines.xhtml

Information sources

We will search the following databases for relevant articles from inception to March 2020: Medline, Embase, Global Health, Scopus, Cochrane Library (which includes Cochrane Reviews, Cochrane Protocols, Trials, Editorials, Special Collections, Clinical Answers and Other Reviews), Web of Science (which includes the Science Citation Index Expanded [SCI-EXPANDED]; the Social Sciences Citation Index [SSCI]; the Arts & Humanities Citation Index [A&HCI]; the Conference Proceedings Citation Index-Science [CPCI-S]; the Conference Proceedings Citation Index – Social Science & Humanities [CPCI-SSH]; and the Emerging Sources Citation Index [ESCI]), Base, PsycInfo and the Global Resource of Eczema Trials (GREAT). Both Medline and Embase capture a large amount of published literature – Medline indexes more than 5,200 journals and Embase indexes almost 8,500 journals,[22,23] – while the other databases are likely to contain appropriate papers for this review. To ensure that all relevant literature is included in the review, we will also search for grey literature in Open Grey, the New York Academy of Medicine Grey Literature Report and PsycExtra. Finally, we will search the five largest clinical trial registries – ClinicalTrials.gov, the EU Clinical Trials Register, the Japan Primary Registries Network (JPRN), ISRCTN and the Australian New Zealand Clinical Trials Registry (ANZCTR) – to identify relevant trials.[24]

Search strategy

We will search medical subject headings and free text (in titles, abstracts and keywords) for synonyms relating to three key concepts: (1) ‘risk factors’, (2) ‘atopic

For peer review only - http://bmjopen.bmj.com/site/about/guidelines.xhtml

eczema or psoriasis' and (3) 'mental illness' (**Table 2**). We will combine the three key concepts in the search strategy using the Boolean logic operator 'AND'. We have developed and piloted an initial search strategy in the Medline database that has been peer reviewed by a librarian (**Supplementary Table 1**), and we will adapt it appropriately for other databases. We will also manually scrutinise the reference lists and bibliographies of relevant systematic reviews to identify additional papers for inclusion. Finally, we will use citation searching on large summary papers to identify any further relevant publications.

For peer review only

For peer review only - http://bmjopen.bmj.com/site/about/guidelines.xhtml

Table 2: Keywords included in the search strategy for all databases

Search term	Keywords
Risk factor terms	risk **OR** risk factor* **OR** protective factor **OR** predict* **OR** correlat* **OR** associat* **OR** aetiol* **OR** etiol* **OR** relationship **OR** mediat* **OR** mechanism* **OR** caus* **OR** path*
Atopic eczema or psoriasis terms	atopic dermatitis **OR** eczema **OR** atopy **OR** psoriasis **OR** psoria* **OR** (pustulo* AND palmopl* OR palmari* OR palmar)
Mental illness terms	mental health **OR** mental* ill* **OR** mental disorder* **OR** psychiatr* ill* **OR** psychiatr* disorder **OR** psychiatr* disease* **OR** psychological* ill* **OR** psychological* disorder* **OR** psychological* disease* **OR** affective* **OR** anxi* **OR** depress* **OR** phobi* **OR** panic **OR** bipolar* **OR** schizophrenia **OR** schizo* **OR** delusion* **OR** psychotic* **OR** psychos#s **OR** psychological* distress

For peer review only

Study records

Data management

A single reviewer (EA) will import all results returned from the electronic database searches into the reference management tool EndNote X9 (Clarivate Analytics, V.9.2/2019). After identifying and removing duplicate records, we will import the search results into Rayyan (a web application for systematic reviews),[25] where the integrated deduplication function will be used to identify any previously missed duplicates.

Study selection

Two reviewers (EA and YS) will independently screen the titles and abstracts of the search results for potentially relevant studies. Both reviewers will then screen the full text of all potentially relevant studies for inclusion using the eligibility criteria. Any disagreements during this process will be discussed by EA and YS, with consultation from a third reviewer (KM) if necessary. We will record and report in a flowchart the reasons for study rejection following full text screening.

Data extraction

We will develop a standardised data extraction form (to extract information described below), which will be piloted by two reviewers (EA and YS) who will extract data from the larger of either 10% or five of the eligible studies. Any disagreements between the two reviewers will be discussed, with a third reviewer (KM) available to arbitrate if required, and changes made to the data extraction form if necessary. A single

For peer review only - http://bmjopen.bmj.com/site/about/guidelines.xhtml

reviewer (EA) will complete the extraction of data for the remaining studies. We will use a modified version of the PICOS (Population, Intervention, Comparator(s), Outcome(s) and Study Design) framework to summarise data for extraction.[26] However, due to the inclusion of observational studies in our review, we will replace the term ‘intervention’ with ‘exposure’, and ‘study design’ will be replaced by ‘study characteristics’. We will extract information for each component of the PICOS framework, in addition to study results for each study included in the review (**Table 3**).

For peer review only

Table 3: Items that will be collected using the data extraction form

Parameter	Information for extraction
Population	Participant inclusion and exclusion criteria<br>Demographic characteristics (age, sex and ethnicity distributions)<br>Sample size
Exposure	Definition and identification of individuals with the risk factor(s) of interest<br>Number of individuals with the risk factor(s) of interest
Comparator	Definition and identification of individuals without the risk factor(s) of interest<br>Number of individuals without the risk factor(s) of interest
Outcome	Definition and identification of mental illness outcome(s)<br>Number of individuals in exposed and comparison group with the outcome
Study characteristics	Bibliographic information (authors, journal, publication year, volume, page numbers, doi)<br>Study design<br>Study setting<br>Study sampling frame<br>Methods of participant recruitment<br>Aims and objectives
Study results	Unadjusted and fully adjusted effect estimates for the association between risk factors and mental illness<br>Confounders measured and adjusted for in analysis

For peer review only

For peer review only - http://bmjopen.bmj.com/site/about/guidelines.xhtml

Exposures

Our exposures of interest will be risk factors for mental illness in people with atopic eczema or psoriasis. We will consider any variable that authors of included papers have conducted analyses to assess whether they are associated with mental illness in people with atopic eczema or psoriasis as potential risk factors. These may include sociodemographic factors (e.g. sex, ethnicity, deprivation), lifestyle factors (e.g. level of physical activity, diet, alcohol consumption) or environmental factors.

Outcomes

Our primary outcome of interest will be mental illness in individuals with atopic eczema or psoriasis. Mental illness will be grouped into two broad categories (CMD or SMI), unless there are sufficient studies looking at specific mental illnesses (e.g. depression) when we will also explore by specific mental illness subgroup. We will include studies regardless of how they capture mental illness outcomes (i.e. we will include clinical diagnoses or self-reported mental illness established with or without validated tools).

Risk of bias assessment for individual studies

Two reviewers (EA and YS) will independently assess the risk of bias for the larger of 10%, or five, of the included studies. Any disagreements will be discussed so that a consensus can be reached. A third reviewer (KM) will be available to arbitrate if required. A single reviewer (EA) will then assess risk of bias for the remaining studies. We will use the Quality in Prognosis Studies (QUIPS) tool to assess the risk

For peer review only

For peer review only - http://bmjopen.bmj.com/site/about/guidelines.xhtml

of bias of included studies.[27] QUIPS assesses and evaluates the risk of bias in six different domains: (1) study participation; (2) study attrition; (3) prognostic factor measurement; (4) outcome measurement; (5) study confounding; and (6) statistical analysis and reporting.[27] For each study included, we will assess and categorise the risk of bias for each domain into one of three qualitative categories (low, moderate or high risk of bias) using the prompting items provided within the tool. We will produce separate risk of bias tables for observational studies and RCTs, along with justifications for the decisions made.

For peer review only

Data synthesis and meta-bias(es)

We will synthesise our results narratively. We will describe and tabulate the results of the studies included in the review according to the study design (RCT, cohort, case-control or cross-sectional study), skin disease type (either atopic eczema or psoriasis), risk factor under investigation and outcome measure (either CMD or SMI). We will describe and tabulate the results of the randomised controlled trials separately from the results of other studies included in the review. If possible, we will also identify risk factors that are common and distinct between atopic eczema and psoriasis. If at least two studies are sufficiently homogeneous (in terms of study design, study population, risk factor assessed and outcome), we will consider a meta-analysis to pool the effect estimates. We will use the I^2 statistic to quantify levels of statistical heterogeneity (I^2 of 0-40% may indicate negligible heterogeneity, 30-60% may indicate moderate heterogeneity, 50-90% may indicate substantial heterogeneity and 75-100% may indicate considerable heterogeneity).[24] If possible, we will also consider meta-regression to investigate whether study characteristics (e.g. study design, risk of bias, study outcome, skin disease) or the demographics of the study population (e.g. age and sex) are associated with the magnitude of effects and can explain any observed statistical heterogeneity. We will assess the risk of publication bias for the studies included in the review using funnel plots. We will use STATA V.16.0 to perform all statistical analysis.

Confidence in cumulative evidence

For peer review only - http://bmjopen.bmj.com/site/about/guidelines.xhtml

We will use the Grading of Recommendations, Assessment, Development and Evaluation (GRADE) framework to evaluate and summarise the quality of cumulative evidence for each broad outcome (CMD or SMI) and risk factor pair.[28] If more than one study is identified for a specific subtype of a CMD or SMI (such as depression or schizophrenia) and a specific risk factor, we will use GRADE to summarise the quality of evidence for that subtype. We will categorise the strength of evidence into four qualitative categories: 'high', 'moderate', 'low' or 'very low'. The quality of evidence for included studies will be upgraded if there is a large magnitude of effect or a dose-response gradient.[28] The quality of evidence will be rated down if there is a high risk of bias, imprecision in the study estimate, a high probability of publication bias or inconsistent results.[28] We will present the judgments made during this process in a 'Summary of Findings' table.

For peer review only

ETHICS AND DISSEMINATION

As this study is a systematic review that does not involve human participation, we do not require ethical approval. We will disseminate the results of this review by publishing in an open access, peer-reviewed journal and presenting at conferences. We will document any important amendments and protocol deviations, along with justifications, and publish them as an appendix in the final review.

For peer review only

REFERENCES

1. Parisi R, Symmons DP, Griffiths CE et al. Global Epidemiology of Psoriasis: A Systematic Review of Incidence and Prevalence. *J Invest Dermatol*. 2013; 133(2):377-85. doi: 10.1038/jid.2012.339
2. Barbarot S, Auziere S, Gadkari A et al. Epidemiology of atopic dermatitis in adults: Results from an international survey. *Allergy*. 2018;73(6):1284-1293. doi: 10.1111/all.13401
3. James S, Abate D, Abate K et al. Global, regional, and national incidence, prevalence, and years lived with disability for 354 diseases and injuries for 195 countries and territories, 1990–2017: a systematic analysis for the Global Burden of Disease Study 2017. *Lancet*. 2018;392(10159):1789-1858. doi: 10.1016/S0140-6736(18)32279-7
4. NHS Digital. *Adult Psychiatric Morbidity Survey: Survey of Mental Health and Wellbeing, England, 2014*. Available from: https://digital.nhs.uk/data-and-information/publications/statistical/adult-psychiatric-morbidity-survey/adult-psychiatric-morbidity-survey-survey-of-mental-health-and-wellbeing-england-2014#key-facts [Accessed 21st January 2020]
5. Chesney E, Goodwin G, Fazel S. Risks of all-cause and suicide mortality in mental disorders: a meta-review. *World Psychiatry*. 2014;13(2):153-160. doi: 10.1002/wps.20128.
6. Eckert L, Gupta S, Amand C et al. Impact of atopic dermatitis on health-related quality of life and productivity in adults in the United States: An analysis using the National Health and Wellness Survey. *J Am Acad Dermatol*. 2017;77:274–279.e3. doi: 10.1016/j.jaad.2017.04.019
7. Klokk M, Gotestam KG, Mykletun A. Factors accounting for the association between anxiety and depression, and eczema: The Hordaland health study (HUSK). *BMC Dermatol*. 2010;10:3. doi: 10.1186/1471-5945-10-3
8. Schmitt J, Romanos M, Pfennig A et al. Psychiatric comorbidity in adult eczema. *Br J Dermatol*. 2009;161(4):878–83. doi: 10.1111/j.1365-2133.2009.09309

9. Ferreira BR, Pio-Abreu JL, Reis JP et al. Analysis of the prevalence of mental disorders in psoriasis: The relevance of psychiatric assessment in dermatology. *Psychiatr Danub*. 2017;29(4):401–6. doi: 10.24869/psyd.2017.401.

10. Kurd SK, Troxel AB, Crits-Cristoph P et al. The Risk of Depression, Anxiety, and Suicidality in Patients With Psoriasis. *Arch Dermatol*. 2010;146(8):891–5. doi: 10.1001/archdermatol.2010.186

11. Tu HP, Yu CL, Lan CCE et al. Prevalence of schizophrenia in patients with psoriasis: a nationwide study. *Dermatologica Sin*. 2017;35(1):1–6. doi: 10.1016/j.dsi.2016.07.005

12. Schonmann Y, Mansfield K, Hayes J et al. Atopic Eczema in Adulthood and Risk of Depression and Anxiety: A Population-Based Cohort Study. *J Allergy Clin Immunol Pract*. 2020;8(1):248-257. doi: 10.1016/j.jaip.2019.08.030

13. Drucker AM, Thiruchelvam D, Redelmeier DA. Eczema and subsequent suicide: a matched case–control study. *BMJ Open*. 2018;8:e023776. doi: 10.1136/bmjopen-2018-023776

14. Sandhu JK, Wu KK, Bui T et al. Association Between Atopic Dermatitis and Suicidality: A Systematic Review and Meta-analysis. *JAMA Dermatol.* 2019;155(2):178–187. doi:10.1001/jamadermatol.2018.4566

15. Bao Q, Chen L, Lu Z et al. Association between eczema and risk of depression: A systematic review and meta-analysis of 188,495 participants*. J Affect Disord*. 2018; 238:458–64.doi: 10.1016/j.jad.2018.05.007

16. Rønnstad ATM, Halling-Overgaard A-S, Hamann CR et al. Association of atopic dermatitis with depression, anxiety, and suicidal ideation in children and adults: A systematic review and meta-analysis. *J Am Acad Dermatol*. 2018;79(3):448-456.e30. doi: 10.1016/j.jaad.2018.03.017

17. Dowlatshahi EA, Wakkee M, Arends LR et al. The prevalence and odds of depressive symptoms and clinical depression in psoriasis patients: A systematic review and meta-analysis. *J Invest Dermatol*. 2014;134(6):1542–1551. doi: 10.1038/jid.2013.508

For peer review only - http://bmjopen.bmj.com/site/about/guidelines.xhtml

18. Ferreira B.I.R.C, Abreu J.L.P., Dos Reis J.P.G. et al. Psoriasis and associated psychiatric disorders: A systematic review on etiopathogenesis and clinical correlation. *J Clin Aesthet Dermatol*. 2016;9(6):36–43. Available from: https://www.ncbi.nlm.nih.gov/pmc/articles/PMC4928455/ [Accessed 21st January 2020]

19. Patel K, Immaneni S, Singam V et al. Association between atopic dermatitis, depression, and suicidal ideation: A systematic review and meta-analysis. *J Am Acad Dermatol*. 2019; 80(2):402-410. doi: 10.1016/j.jaad.2018.08.063

20. Xie Q, Dai X, Tang X et al. Risk of Mental Disorders in Children and Adolescents with Atopic Dermatitis: A Systematic Review and Meta-Analysis. *Front Psychol*. 2019; 10:1773. doi: 10.3389/fpsyg.2019.01773

21. Moher D, Shamseer L, Clarke M et al. Preferred reporting items for systematic review and meta-analysis protocols (PRISMA-P) 2015 statement. *Syst Rev*. 2015;4(1):1. doi: https://doi.org/10.1186/2046-4053-4-1

22. U.S National Library of Medicine. *Fact sheet: MEDLINE*. Available from: https://www.nlm.nih.gov/lstrc/jsel.html. [Accessed 27th January 2020]

23. Elsevier. *EMBASE: Coverage and Content*. Available from: https://www.elsevier.com/solutions/embase-biomedical-research/embase-coverage-and-content. [Accessed 27th January 2020]

24. Huser V, Cimino JJ. Evaluating adherence to the International Committee of Medical Journal Editors' policy of mandatory, timely clinical trial registration. J Am Med Inform Assoc. 2013; 20(e1): e169-e174. doi: 10.1136/amiajnl-2012-001501

25. Ouzzani M, Hammady H, Fedorowicz Z et al. Rayyan – a web and mobile app for systematic reviews. Syst Rev. 2016;5(1):210. doi: 10.1186/s13643-016-0384-4

26. Higgins J, Green S. *Cochrane Handbook for Systematic Reviews of Interventions: Version 5.1.0 (updated March 2011)*. 2011. Available from: www.handbook-5-1.cochrane.org [Accessed 21st January 2020]

For peer review only - http://bmjopen.bmj.com/site/about/guidelines.xhtml

27. Hayden JA, van der Windt DA, Cartwright JL et al. Assessing bias in studies of prognostic factors. *Ann Intern Med*. 2013; 158(4):280-286. doi: 10.7326/0003-4819-158-4-201302190-00009

28. Guyatt G, Oxman A, Vist G et al. GRADE: an emerging consensus on rating quality of evidence and strength of recommendations. BMJ. 2008; 336(7650):924-6. doi: 10.1136/bmj.39489.470347.AD

For peer review only

For peer review only - http://bmjopen.bmj.com/site/about/guidelines.xhtml

ACKNOWLEDGEMENTS AND DECLARATIONS

Contributions

EA, SL and KM had the original idea for the review. All authors (EA, YS, JH, RM, AM, LR, LS, CH, SL and KM) were involved in the design of the study. EA wrote the first draft of the protocol. All authors (EA, YS, JH, RM, AM, LR, LS, CH, SL and KM) contributed to further drafts and approved the final manuscript. Kate Perris peer reviewed the search strategy.

Funding

EA was funded by a British Skin Foundation (BSF) PhD studentship (Reference: 024/S/18). SML was funded by a Wellcome Trust Senior Clinical Fellowship in Science (Reference: 205039/Z/16/Z).

Competing interests

None declared.

For peer review only

Supplementary Material

Supplementary Table 1: Search strategy in MEDLINE database

Item number	Searches
<u>Risk factor terms</u>	
1	risk OR risk factor* OR protective factor OR predict* OR correlat* OR associate* OR aetiol* OR etiol* OR relationship OR mediat* OR mechanism* OR caus* OR path*
2	exp Risk/
3	1 OR 2
<u>Atopic eczema terms</u>	
4	atopic dermatitis OR atopic eczema OR atopy
5	Dermatitis, Atopic/
6	exp Eczema/
7	4 OR 5 OR 6
<u>Psoriasis terms</u>	
8	psoriasis OR psoria*
9	pustulo* AND (palmopl* OR palmari* OR palmar)
10	exp Psoriasis/
11	8 OR 9 OR 10
<u>Combining atopic eczema and psoriasis terms with 'OR'</u>	
12	7 OR 11
<u>Mental illness terms</u>	
13	mental health OR mental* ill* OR mental disorder* OR affective OR anxi* OR depress* OR phobi* OR panic OR bipolar* OR schizo* OR schizophrenia OR delusion* OR psychotic* OR psychos#s
14	psychiatr* AND (ill* OR disorder OR disease*)
15	psychological* AND (ill* OR disorder OR disease* OR distress)
16	Mental Health/
17	Exp Mental Disorders/
18	13 OR 14 OR 15 OR 16 OR 17
<u>Combining key concepts with 'AND'</u>	
19	3 AND 12 AND 18

For peer review only

For peer review only - http://bmjopen.bmj.com/site/about/guidelines.xhtml

Reporting checklist for protocol of a systematic review.

Based on the PRISMA-P guidelines.

Instructions to authors

Complete this checklist by entering the page numbers from your manuscript where readers will find each of the items listed below.

Your article may not currently address all the items on the checklist. Please modify your text to include the missing information. If you are certain that an item does not apply, please write "n/a" and provide a short explanation.

Upload your completed checklist as an extra file when you submit to a journal.

In your methods section, say that you used the PRISMA-Preporting guidelines, and cite them as:

Moher D, Shamseer L, Clarke M, Ghersi D, Liberati A, Petticrew M, Shekelle P, Stewart LA. Preferred Reporting Items for Systematic Review and Meta-Analysis Protocols (PRISMA-P) 2015 statement. Syst Rev. 2015;4(1):1.

		Reporting Item	Page Number
**Title**			
Identification	#1a	Identify the report as a protocol of a systematic review	1
Update	#1b	If the protocol is for an update of a previous systematic review, identify as such	n/a

For peer review only

**Registration**			
	#2	If registered, provide the name of the registry (such as PROSPERO) and registration number	2
**Authors**			
Contact	#3a	Provide name, institutional affiliation, e-mail address of all protocol authors; provide physical mailing address of corresponding author	1
Contribution	#3b	Describe contributions of protocol authors and identify the guarantor of the review	18
**Amendments**			
	#4	If the protocol represents an amendment of a previously completed or published protocol, identify as such and list changes; otherwise, state plan for documenting important protocol amendments	14
**Support**			
Sources	#5a	Indicate sources of financial or other support for the review	18
Sponsor	#5b	Provide name for the review funder and / or sponsor	18
Role of sponsor or funder	#5c	Describe roles of funder(s), sponsor(s), and / or institution(s), if any, in developing the protocol	18
**Introduction**			

For peer review only

Rationale	#6	Describe the rationale for the review in the context of what is already known	4-5
Objectives	#7	Provide an explicit statement of the question(s) the review will address with reference to participants, interventions, comparators, and outcomes (PICO)	4-5
**Methods**			
Eligibility criteria	#8	Specify the study characteristics (such as PICO, study design, setting, time frame) and report characteristics (such as years considered, language, publication status) to be used as criteria for eligibility for the review	7 (Table 1)
Information sources	#9	Describe all intended information sources (such as electronic databases, contact with study authors, trial registers or other grey literature sources) with planned dates of coverage	8
Search strategy	#10	Present draft of search strategy to be used for at least one electronic database, including planned limits, such that it could be repeated	8-9, Table 2 and Supplementary table 1
Study records - data management	#11a	Describe the mechanism(s) that will be used to manage records and data throughout the review	10
Study records - selection process	#11b	State the process that will be used for selecting studies (such as two independent reviewers)	10

For peer review only

		through each phase of the review (that is, screening, eligibility and inclusion in meta-analysis)	
Study records - data collection process	#11c	Describe planned method of extracting data from reports (such as piloting forms, done independently, in duplicate), any processes for obtaining and confirming data from investigators	10
Data items	#12	List and define all variables for which data will be sought (such as PICO items, funding sources), any pre-planned data assumptions and simplifications	11 (Table 3)
Outcomes and prioritization	#13	List and define all outcomes for which data will be sought, including prioritization of main and additional outcomes, with rationale	12
Risk of bias in individual studies	#14	Describe anticipated methods for assessing risk of bias of individual studies, including whether this will be done at the outcome or study level, or both; state how this information will be used in data synthesis	12
Data synthesis	#15a	Describe criteria under which study data will be quantitatively synthesised	13
Data synthesis	#15b	If data are appropriate for quantitative synthesis, describe planned summary measures, methods of handling data and methods of combining data from studies, including any planned exploration of consistency (such as I2, Kendall's τ)	13

For peer review only

Data synthesis	#15c	Describe any proposed additional analyses (such as sensitivity or subgroup analyses, meta-regression)	13
Data synthesis	#15d	If quantitative synthesis is not appropriate, describe the type of summary planned	13
Meta-bias(es)	#16	Specify any planned assessment of meta-bias(es) (such as publication bias across studies, selective reporting within studies)	13
Confidence in cumulative evidence	#17	Describe how the strength of the body of evidence will be assessed (such as GRADE)	13

None The PRISMA-P checklist is distributed under the terms of the Creative Commons Attribution License CC-BY 4.0. This checklist can be completed online using https://www.goodreports.org/, a tool made by the EQUATOR Network in collaboration with Penelope.ai

For peer review only
